# The Relationship Between Avoidant/Restrictive Food Intake Disorder in Children Diagnosed with Autism Spectrum Disorder and Orthorexia Nervosa in Their Mothers

**DOI:** 10.1007/s10803-025-06818-8

**Published:** 2025-04-17

**Authors:** Borte Gurbuz Ozgur, Buket Canlan Ozaydin, Rabia Eren, Ufuk Uyar, Yigit Ozaydin, Hatice Aksu

**Affiliations:** 1 Department of Child and Adolescent Psychiatry, Aydın Adnan Menderes University Medical Faculty, Aydın, 09100 Türkiye; 2 Department of Child and Adolescent Psychiatry, Dr. Behcet Uz Pediatric Diseases and Surgery Training and Research Hospital, Izmir, Türkiye; 3 Department of Child and Adolescent Psychiatry, Ege University Medical Faculty, Izmir, Türkiye; 4 Department of Child and Adolescent Psychiatry, Izmir Tınaztepe University Medical Faculty, Izmir, Türkiye

**Keywords:** Autism spectrum disorder, Avoidant restrictive food intake disorder, Orthorexia nervosa, Sensory sensitivity

## Abstract

The aim is to examine the relationship between Avoidant/Restrictive Food Intake Disorder (ARFID) symptoms and sensory sensitivities in children diagnosed with Autism Spectrum Disorder (ASD), as well as the relationship between maternal orthorexia nervosa (ON) and ARFID, and to identify the factors influencing ARFID. The symptom severity of 104 children was assessed using the Childhood Autism Rating Scale (CARS), maternal ON symptoms with ORTO-11, ARFID symptoms with the Nine-Item Avoidant/Restrictive Food Intake Disorder Screening Tool (NIAS), and sensory sensitivities with the Eyuboglu Sensory Reactivity Scale (ESRS). Multiple regression analyzed predictors of NIAS scores, and moderator analysis examined whether ORTO-11 moderated the ESRS-NIAS relationship. ON was present in 58% of the mothers. Mothers with ON had significantly higher total NIAS scores and NIAS Fear subscale scores. A positive and statistically significant relationship was found between the CARS scores and the hyporeactivity and sensory-seeking subscales of the ESRS scale. When NIAS was taken as the dependent variable, a significant regression relationship was found between CARS-9 and ORTO-11. However, ORTO-11 does not play a moderating role in the effect of ESRS on NIAS. ARFID symptoms are predicted by maternal ON symptoms and CARS-9 scores in children. We emphasize the importance of evaluating the eating attitudes and food perspectives of caregivers when atypical eating behaviors are identified in the clinical follow-up of children diagnosed with ASD. Since the study was conducted solely with mothers’, further research is needed to examine the effects of ON symptoms in fathers and other caregivers.

## Introduction

Autism spectrum disorder (ASD) is a neurodevelopmental disorder characterized by deficiencies in social communication and social interaction, accompanied by restricted interests and repetitive behaviors. According to DSM-5 (Diagnostic and Statistical Manual of Mental Disorders), for a diagnosis of ASD, all three criteria in the ‘deficits in social interaction/communication’ domain and at least two of the four criteria in the ‘restricted, repetitive interests, behaviors, and activities’ domain must be met (American Psychiatric Association. DSM-5 Task Force., 2013).

Recent comprehensive population screening studies have shown an increase in the prevalence of ASD. A 2018 study conducted in the United States suggested that the prevalence of ASD could be found in 1 in 36 children (Maenner et al., 2023). A recent study in our country involving 6,712 children aged 16–36 months found the prevalence of ASD to be 1/117 (Oner & Munir, 2020). The literature reports that over 70% of ASD cases are accompanied by simultaneous medical, developmental, or psychiatric comorbid conditions. Although it is often seen alongside other neurodevelopmental disorders, conditions such as depression, sleep problems, gastrointestinal issues, and eating disorders may also accompany ASD (Lai et al., 2014).

Eating is a fundamental activity that supports growth and sustains life. For infants, feeding is a complex skill that develops during the first two years of life (Satter, 2007). A healthy feeding relationship encompasses both the child’s physiological needs and the emotional aspect of the parent’s feeding responsibility (Delaney & Arvedson, 2008). Possessing healthy eating skills can make mealtimes a source of satisfaction for both the child and the parent, whereas feeding problems can be a significant source of stress for both (Vissoker et al., 2015). In addition, children with ASD are more likely than other children to experience feeding issues such as food refusal, specific mealtime behaviors, and acceptance of a limited variety and texture of foods (Schreck et al., 2004).

Avoidant/Restrictive Food Intake Disorder (ARFID) is a diagnosis newly added to DSM-5, characterized by reluctance or selective eating. According to DSM-5, the primary diagnostic criteria for ARFID include a lack of interest in food or eating, avoidance based on the sensory characteristics of food (such as smell or appearance), or concern about the aversive consequences of eating (such as choking or vomiting). As a result, one or more of the following must be present: significant weight loss, nutritional deficiency, dependence on nutritional supplements, or marked interference with psychosocial functioning (American Psychiatric Association. DSM-5 Task Force., 2013).

Additionally, ARFID has been used clinically to describe restrictive eating behaviors that are not driven by body image disturbances or fear of weight gain. The three features commonly observed in clinical practice are: a clear lack of interest in food (low appetite, no pleasure from eating), avoidance based on the sensory characteristics of food (sensory sensitivity), and/or concerns about the negative consequences of eating (choking, vomiting). Research to fully understand its etiology is ongoing, though, like other eating disorders, biological and environmental factors are thought to be involved (Bourne et al., 2022).

A review of the literature shows that epidemiological studies on ARFID are limited. One study reported that 5–22% of children and adolescents seeking evaluation for eating disorders or receiving treatment for feeding problems met the diagnostic criteria for ARFID (Nicely et al., 2014). Although population-based prevalence studies based on clinical evaluations are not yet available, a self-report survey of primary school children in Switzerland found the overall prevalence of ARFID to be 3.5% (Thomas et al., 2017). No research on the prevalence of ARFID in Türkiye has been found in the literature.

Studies report that ARFID often co-occurs with several psychiatric conditions, including ASD, attention deficit hyperactivity disorder (ADHD), and anxiety disorders (Zimmerman & Fisher, 2017). Despite the growing body of literature on ARFID in both clinical and general populations, there is limited research on the co-occurrence of ARFID and ASD. In a 2021 study, Koomar et al. found the co-occurrence of ASD and ARFID to be 22% (Koomar et al., 2021).

In the literature, although three categories that cause eating and restriction in children and adolescents with ASD within ARFID have been identified, the most frequently cited is sensory sensitivity (Bourne et al., 2022). Sensory processing refers to the ability to receive, organize, and interpret stimuli, including verbal, visual, tactile, vestibular, and auditory experiences. Processing issues can lead to difficulties in interpreting sensory inputs and result in abnormal behavioral patterns (Germani et al., 2014; Panerai et al., 2020). Sensory processing issues, as a symptom of ASD, may manifest as hypo- or hyper-responsiveness to sensory input, and may also be reflected in food selectivity. Children with ASD may experience food-related issues, such as avoidance of certain foods, textures, tastes, smells, and temperatures (Cermak et al., 2010).

The relationship between children and parents during the feeding process can be a significant factor in the development of eating problems. Children’s eating habits are shaped by factors such as exposure to and accessibility of food, parenting style, the choice of feeding methods, and the modeling behaviors of siblings and parents (Ventura & Birch, 2008). One of the heightened concerns for parents of children with any illness is ensuring proper care, which often includes providing a healthy diet. Parents monitor whether their children are eating healthily by restricting unhealthy foods. It is expected that individuals with a tendency towards an obsession with healthy eating will feed their children in a similar manner.

Since the mother typically plans and prepares meals at home, her dietary habits have a direct impact on the child’s nutrition. Additionally, women tend to make healthier food choices and show greater awareness about diet (Boutelle et al., 2007).

Research indicates that children with ASD often exhibit a marked reluctance to try new foods, leading to a restricted diet that may consist of only a few preferred items (Cermak et al., 2010; Mari-Bauset et al., 2014; Marshall et al., 2014). This phenomenon is not unique to ASD; rather, food selectivity is a behavior observed across various populations, including neurotypical children, albeit with differing degrees of severity (Amin et al., 2022; Byrska et al., 2023). Studies have shown that while children with ASD display more pronounced food preferences and aversions, similar selective eating behaviors can also be found in typically developing children, suggesting that food restriction is a common aspect of childhood development (Amin et al., 2022; Byrska et al., 2023; Cermak et al., 2010). Furthermore, the sensory sensitivities associated with ASD often exacerbate these selective eating patterns, as many children prefer specific textures, colors, or tastes, which can lead to nutritional deficiencies if not properly managed (Graf-Myles et al., 2013; Xia et al., 2010).

Orthorexia nervosa (ON) is a relatively new concept in the field of eating disorders. Individuals with orthorexia exhibit a “maniacal obsession” in the pursuit of healthy foods (Donini et al., 2004). However, ON is not yet included in the DSM-5, the proposed criteria for ON include compulsive behavior and/or mental preoccupation regarding affirmative and restrictive dietary practices believed by the individual to promote optimum health; violation of self-imposed dietary rules causes exaggerated fear of disease, sense of personal impurity and/or negative physical sensations, accompanied by anxiety and shame; dietary restrictions escalate over time; malnutrition, severe weight loss or other medical complications from restricted diet; intrapersonal distress or impairment of social, academic or vocational functioning secondary to beliefs or behaviors about healthy diet; positive body image, self-worth, identity and/or satisfaction excessively dependent on compliance with self-defined “healthy” eating behavior. (Dunn & Bratman, 2016; Koven & Abry, 2015).

Although there is no definitive data on the prevalence of this disorder, research on ON has increased in medical literature. In a study by Donini et al., the prevalence of ON was reported to be 6.9% (Donini et al., 2004). In another study conducted by Ramacciotti et al. in Italy, the diagnostic criteria for ON were met by 57.6% of a sample of 107 individuals (Ramacciotti et al., 2011). A study conducted in Türkiye with 318 resident physicians found a prevalence rate of 45.5% (Bagci Bosi et al., 2007).

Nutritional management in children with ASD is influenced not only by biological but also psychosocial and environmental factors. It is frequently emphasized in the literature that feeding problems in children with ASD are associated with sensory sensitivity, limited food preferences and behavioural problems during meals (Brzoska et al., 2021; Elshafie Elnajjar, 2021). The roles of parents and caregivers in the feeding process are critical in shaping the eating habits of children with ASD (Lazaro & Ponde, 2017). In the context of maternal orthorexia, it is essential to explore how long mothers have struggled with this condition, the impact of their dietary behaviors on family dynamics and quality of life, and their emotional experiences when their children receive an ASD diagnosis. Understanding these factors can provide deeper insight into the interplay between ON and maternal stressors.

Additionally, the study seeks to identify factors influencing ARFID. While maternal concerns regarding healthy nutrition and dietary control are significant, other emotional and psychological processes may also play a crucial role in shaping these behaviors. Beyond individual preferences, socioeconomic and demographic characteristics of families play a fundamental role in shaping eating habits. Studies have shown that the economic status, education level, region of residence and family structure of individuals have determinant effects on food diversity, healthy diet quality and growth indicators of children (İnan et al., 2024; Ozen et al., 2024). In studies conducted in Türkiye, it has been reported that low-income families have difficulty in accessing fresh and varied foods, mothers’ education level directly affects children’s eating behaviours, and eating habits show regional differences (Ozen et al., 2024; Öz & Bayhan, 2021). In addition, Orün et al. reported that problematic eating behaviours were prevalent in approximately 40% of Turkish children aged 12–72 months and that this was associated with inadequate consumption of meat, vegetables and fruits (Orun et al., 2012). These factors could interact with their children’s dietary behaviors, necessitating a more comprehensive approach to understanding how maternal experiences influence ARFID development in children with ASD.

A review of the literature shows that most studies on the tendency toward healthy eating obsession have been conducted with individuals working or studying in the fields of health or nutrition. No studies have been found examining mothers of children with neurodevelopmental disorders. Understanding the complex relationship between children’s eating habits and their mothers’ eating obsessions could be an important step toward improving the quality of life for both children and families.

In this study, the aim is to investigate the relationship between the ARFID symptoms and sensory sensitivities of individuals with ASD, as well as the relationship between ON in their mothers and ARFID, and to identify the factors influencing ARFID.

## Methods

### Participants

Between December 1, 2023, and January 31, 2024, mothers of male and female patients aged 2–18 years who were being followed up with an ASD diagnosis at the Aydın Adnan Menderes University Child and Adolescent Psychiatry Department and who agreed to participate were consecutively included in the study. The diagnosis of ASD was made by competent child psychiatry specialists through psychiatric evaluation using the Childhood Autism Rating Scale (CARS) according to DSM-5. A total of 160 individuals diagnosed with ASD were included in the study. Fifty-six cases with missing data were excluded. In this study, data were obtained directly from mothers as they play a central role in the care and feeding of their children, regardless of their employment status. In Turkish culture, mothers are often the primary caregivers responsible for meal preparation and feeding arrangements, even if they are working outside the home. Therefore, focusing on mothers provides the most direct and relevant insights into the eating habits and nutritional concerns of children with ASD. In line with this, the primary caregivers of the subjects in our study were their mothers and the parents with whom they ate meals during the day.

Approval for the study was obtained from the Non-Interventional Ethics Committee of Aydın Adnan Menderes University (2023/211).

### Instruments

To assess ARFID, parents completed the Nine-Item Avoidant/Restrictive Food Intake Disorder Screening Tool (NIAS). The Eyuboglu Sensory Reactivity Scale (ESRS) was used to evaluate sensory sensitivity. To screen for ON symptoms in parents, the ORTO-11 scale was filled out by the parents.

### Sociodemographic Data Form

This form, created by the authors, was used to determine the sociodemographic and clinical characteristics of the children and adolescents included in the study. Socioeconomic status was classified as income lower than expenditure, equal to or higher than expenditure according to income and expenditure perception.

### Childhood Autism Rating Scale (CARS)

This scale, developed by Schopler and colleagues in 1980, helps distinguish autism from other neurodevelopmental disorders and assess the severity of autism. The total score ranges from 15 to 60. A score of 30-36.5 indicates mild autism, while a score of 37 or higher indicates severe autism (Schopler et al., 1980). The reliability of the total score in terms of test-retest reliability (*r* = 0.98, *p* < 0.01) and interrater reliability (*r* = 0.97, *p* < 0.01) is high, with a cutoff score of 29.5. The Turkish validity and reliability study was conducted by Gassaloğlu (İncekaş-Gassaloğlu et al., 2016).

### ORTO-11

This is a self-report scale developed by Donini et al. (2005) as a shortened version of the original ORTO-15, designed to diagnose orthorexia nervosa (ON). Based on Bratman’s test for orthorexia, some questions were revised, and additional items were included, leading to the creation of the ORTO-15 (Donini et al., 2005). The scale was initially translated into Turkish by Arusoğlu in 2006. However, due to the inability to achieve the desired psychometric properties, Arusoğlu et al. refined the scale, resulting in the current Turkish version known as ORTO-11. In the Turkish adaptation, items 1, 2, 9, and 15 from ORTO-15 were excluded due to insufficient statistical strength. ORTO-11 contains 11 items (questions 3, 4, 5, 6, 7, 8, 10, 11, 12, 13, and 14), with item 8 being reverse scored. Responses are measured on a 4-point Likert scale, from “never” (4) to “always” (1). Lower scores on the ORTO-11 indicate a greater tendency toward orthorexic behavior. The maximum score achievable on the scale is 44. While the Cronbach’s alpha for the original 15-item version was 0.44, the ORTO-11 version has a Cronbach’s alpha of 0.62 (Arusoglu et al., 2008). In line with previous research, this study used the average score of ORTO-11 (27 points) as the cutoff. Scores below this threshold were considered indicative of orthorexic tendencies (Agopyan et al., 2019; Parra-Fernandez et al., 2019). In our study, Cronbach’s alpha was 0.646.

### Nine-Item Avoidant/Restrictive Food Intake Disorder Screening Tool (NIAS)

Developed by Zickgraf and Ellis to screen for ARFID, this parent-completed scale has three subscales: selective eating, lack of appetite/limited interest in eating, and fear of aversive consequences of eating. It consists of nine items rated on a 5-point Likert scale, from “strongly disagree” (0) to “strongly agree” (5) (Zickgraf & Ellis, 2018). Subscale scores range from 0 to 15, with a total score range of 0 to 45. The Turkish adaptation study found a total internal consistency coefficient of 0.90, with subscale consistency coefficients of 0.86, 0.87, and 0.91, respectively (Akçay et al., 2023). In our study, Cronbach’s alpha was 0.859.

### Eyuboglu Sensory Reactivity Scale (ESRS)

Developed by Eyuboglu et al. to measure abnormal sensory reactions and to be used in the follow-up of sensory interventions in children with autism, the ESRS was psychometrically tested for reliability and validity (Eyuboglu et al., 2022). The scale consists of 15 items on a 5-point Likert scale, suitable for children aged 2 to 18. The Cronbach’s alpha value for the scale is 0.85. In our study, Cronbach’s alpha was 0.844.

### Statistical Analysis

The data from the cases were analyzed using IBM SPSS Statistics 24.0 for Windows (SPSS Inc., Chicago, Illinois, USA) and the AMOS Graphics program. Continuous variables were expressed as mean and standard deviation, while categorical variables were expressed as frequency (n) and percentage (%). A statistical significance level of *p* < 0.05 was accepted.

Normality assumptions were evaluated through Skewness and Kurtosis values and histogram charts. The normal distribution of the data was assessed using the Kolmogorov-Smirnov test. In the comparison of scale scores between gender and groups with and without orthorexia nervosa, the Student’s t-test was used for parametric tests, and the Mann-Whitney U test was used for non-parametric tests. The relationships between scale scores were analyzed using Pearson’s correlation test.

**Fig. 1:**
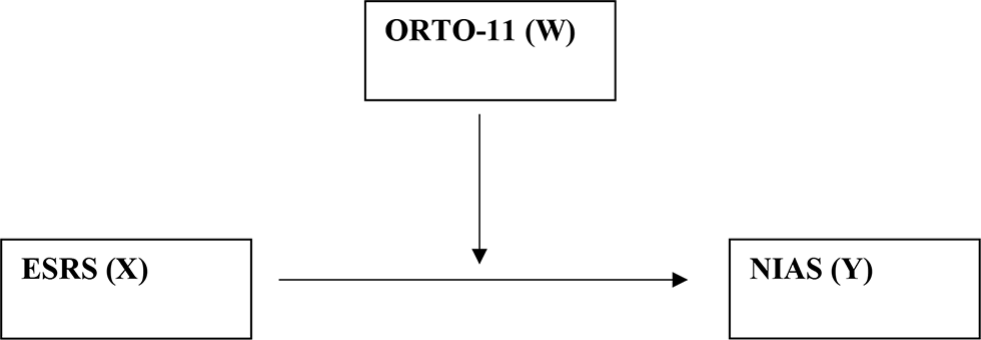
Moderation analysis model. ESRS: The Eyuboglu Sensory Reactivity Scale, NIAS: Nine-Item Avoidant/Restrictive Food Intake Disorder Screening Tool. X = independent variable, Y = dependent variable, W = moderator variable

To test our main hypothesis, linear regression analysis was conducted to determine the factors predicting NIAS. The results include unstandardized beta coefficients and significance values indicating potential relationships. A moderator variable (W) is a variable that influences the direction and strength of the relationship between the predictor variable (X) and the outcome variable (Y) (Baron & Kenny, 1986). In this study, a moderation analysis model was established to investigate whether the effect of the independent variable ESRS (X) on the outcome variable NIAS (Y) is moderated by ORTO-11 (W) (Fig. 1). PROCESS Macro v4.2 model for SPSS was used (Hayes, 2018).

## Results

The mean age of the 104 subjects was 5.92 ± 3.33 years. In terms of gender distribution, 91 (87.5%) were male and 13 (12.5%) were female. The sociodemographic and clinical characteristics of the subjects are presented in Tables 1 and 2.

**Table 1:** Sociodemographic characteristics of the children with ASD

Characteristic	*n*	%
Gender		
- Female	13	12.5
- Male	91	87.5
Education Level		
- Preschool	43	41.3
- Primary School	15	14.2
- Middle School	5	4.8
- High School	2	1.9
- Special Class	6	5.8
School Attendance		
- Currently Attending	71	68.3
- Never Attended	31	29.8
- Dropped Out	2	1.9
Living Arrangement		
- Nuclear Family	98	94.2
- Extended Family	3	2.9
- Only Grandparents	2	1.9
- Only Stepparents	1	1.0
Number of Siblings		
- None	32	30.8
− 1 Sibling	47	45.2
− 2 Siblings	21	20.2
− 3 Siblings	4	3.8
Parental Marital Status		
- Married	98	94.2
- Divorced/Separated	4	3.9
- One Parent Deceased	2	1.9
Mother’s Education Level		
- Illiterate	1	1.0
- Only Literate	1	1.0
- Primary School Graduate	22	21.2
- Middle School Graduate	21	20.2
- High School Graduate	23	22.1
- University Graduate	36	34.6
Mother’s Employment Status		
- Employed	28	26.9
- Unemployed	76	73.1
Father’s Education Level		
- Illiterate	1	1.0
- Primary School Graduate	28	26.9
- Middle School Graduate	19	18.3
- High School Graduate	24	23.1
- University Graduate	32	30.8
Father’s Employment Status		
- Employed	91	87.5
- Unemployed	13	12.5
Income and Expenditure Status		
- Income Less than Expenditure	43	41.3
- Income Equal to Expenditure	35	33.7
- Income More than Expenditure	26	25.0
Parental Psychiatric Illness		
- Mother	9	8.7
- Father	3	2.9
Mother’s Psychiatric Diagnosis		
- Depression	4	3.8
- Anxiety Disorder	3	2.9
- Bipolar Disorder	1	1.0
- Obsessive-Compulsive Disorder	1	1.0
Father’s Psychiatric Diagnosis		
- Anxiety Disorder	2	1.9
- Depression	1	1.0

Nuclear Family: A small family consisting of a mother, father, and unmarried children

Only Literate: A person who can read and write but has not completed primary school

**Table 2:** Clinical characteristics of cases

Variable	*n*	%
Physical Illness	17	16.3
Epilepsy*	8*	7.69
Seasonal Allergy	2	1.9
Familial Mediterranean Fever	1	0.96
Arginase Deficiency*	1*	0.96
Phenylketonuria	1	0.96
Gastroesophageal Reflux	1	0.96
Hypothyroidism*	1*	0.96
Hearing Loss	1	0.96
Cerebral Palsy	1	0.96
Thalassemia Trait	1	0.96
Fatty Acid Oxidation Defect	1	0.96
Psychiatric Diagnosis	14	13.5
Attention Deficit Hyperactivity Disorder	8	7.7
Mild Intellectual Disability	4	3.8
Moderate Intellectual Disability	1	0.96
Conduct Disorder	1	0.96
Medication Use	26	25
Psychotropic Medication*	24	20.19
Metabolic Disease Treatment*	2	1.92
Anti-rheumatic	1	0.96
Psychotropic Medication Type		
Antipsychotic*	17	16.3
Methylphenidate*	7	6.7
Antiepileptic*	6	5.76
Melatonin*	2	1.9
Atomoxetine	1	0.96

*There were comorbid physical illnesses in 2 cases, and polypharmacy in 9 cases

The mean scores of the scales are provided in Table 3. The mean score for the ORTO-11 was 26.46 ± 4.77. When the cut-off score for the ORTO-11 was accepted as 27 points (Agopyan et al., 2019; Parra-Fernandez et al., 2019), 60 individuals (58%) were determined to have orthorexia.

**Table 3:** Mean, standard deviation, and pearson correlation values for the variables

Variables	Mean	SD	1	2	3	4	5	6	7	8	9	10
1. NIAS	18.89	12.20										
2. Selective eating	8.69	5.16	0.865									
3. Lack of appetite/limited interest in eating	6.85	5.00	0.871	0.728								
4. Fear	3.21	4.52	0.687	0.348	0.413							
5.ORTO-11	26.46	4.78	− 0.225*	− 0.173	− 0.119	− 0.216*						
6.CARS	49.76	9.21	0.186	0.057	0.215	0.183	0.052					
7.CARS-9	2.36	0.90	0.323**	0.140	0.240*	0.420**	0.069	0.633				
8.ESRS	23.52	12.14	0.071	− 0.006	0.119	0.080	0.023	0.401**	0.382**			
9. Hyporeactivity	7.93	5.65	0.096	0.022	0.151	0.058	0.003	0.404**	0.384**	0.854		
10. Hyperreactivity	7.91	4.12	0.003	− 0.068	0.020	0.101	− 0.039	0.102	0.132	0.732	0.404	
11. Sensory seeking	7.67	4.98	0.061	0.017	0.104	0.045	0.083	0.434**	0.387**	0.864	0.613	0.498

CARS: Childhood Autism Rating Scale, ESRS: The Eyuboglu Sensory Reactivity Scale, NIAS: Nine-Item Avoidant/Restrictive Food Intake Disorder Screening Tool

Significant at the *<0.05 level, **<0.01 level

In the comparison between the groups with (*n* = 60) and without (*n* = 44) orthorexia, no significant differences were found in the CARS score, CARS Taste, Smell, and Touch Reactions item, NIAS Selective Eating and Lack of appetite/limited interest in eating subscales, and ESRS score. However, there was a significant difference in the NIAS total score (*p* = 0.047) and NIAS Fear subscale between the groups (*p* = 0.001) (Table 4). When comparing groups based on gender, there were no significant differences in the scale values applied to the subjects (*p* > 0.05).

**Table 4:** Comparison of scale scores between mothers with and without orthorexia nervosa

	with ON (*n* = 60, 58%) mean ± SD	without ON (*n* = 44, 42%) mean ± SD	*p*	t/Z value
CARS	48.80 ± 9.16	50.88 ± 9.24	0.209***	-1.258
CARS-9*	2.30 ± 0.97	2.45 ± 0.81	0.259***	-1.129
NIAS-Total	20.95 ± 12.56	15.81 ± 10.76	0.047**	-1.987
Selective eating	9.43 ± 4.99	7.79 ± 5.28	0.113**	1.585
Lack of appetite/limited interest in eating	7.46 ± 5.31	6.00 ± 4.50	0.188**	1.317
Fear	4.05 ± 5.19	2.04 ± 3.13	0.001**	1.700
ESRS-total	22.65 ± 11.95	24.41 ± 12.42	0.399**	− 0.843
Hyporeactivity	7.40 ± 5.58	8.44 ± 5.59	0.371**	− 0.895
Hyperreactivity	7.98 ± 4.16	7.76 ± 4.13	0.893**	0.134
Sensory seeking	7.26 ± 4.99	8.20 ± 5.03	0.294**	-1.049

CARS: Childhood Autism Rating Scale, ON: Orthorexia Nervosa, NIAS: Nine-Item Avoidant/Restrictive Food Intake Disorder Screening Tool

* CARS Taste, Smell, and Touch Reactions item

** Mann-Whitney U test

*** Student T test

In the Pearson correlation analysis conducted, a significant positive correlation was found between the CARS score and the Hyporeactivity subscale of the ESRS (*p* < 0.01, *r* = 0.404). Additionally, a significant positive correlation was observed between the CARS score and the Sensory Seeking subscale of the ESRS (*p* < 0.01, *r* = 0.434) (Table 3).

The linear regression analysis revealed a significant relationship between the CARS-9 Taste, Smell, and Touch Reactions item and the NIAS (*p* < 0.05, *b* = 4.99), as well as a significant regression relationship between the ORTO-11 and NIAS (*p* < 0.05, *b* = -6.34) (Table 5). It was found that sensory sensitivities (measured by ESRS) had no significant effect on ARFID symptoms (measured by NIAS) (b = 0.554, *p* > 0.05), while ON symptoms (measured by ORTO-11) had a significant negative effect (b = − 0.048, *p* < 0.05). The interactional effect (moderating effect) of sensory sensitivity and ON variables (X×W) on ARFID was found to be non-significant (b = − 0.018, *p* = 0.367, CI=[-0.057, 0.021]). The model explains 4.9% of the total variance (R^2^ = 0.049) (Table 6). In other words, ORTO-11 does not play a moderating role in the effect of ESRS on NIAS.

**Table 5:** Results of multiple linear regression analysis when NIAS is the dependent variable (*n* = 104)

Variables	b	Std. Error	ß	*p*
CARS	− 0.014	0.161	− 0.011	0.923
ORTO-11	− 0.634	0.235	− 0.248	0.020
ESRS	− 0.061	0.102	− 0.061	0.575
CARS-9	4.992	1.625	0.370	0.003
Constant	26.03	8.63		0.006

CARS: Childhood Autism Rating Scale, ESRS: The Eyuboglu Sensory Reactivity Scale, NIAS: Nine-Item Avoidant/Restrictive Food Intake Disorder Screening Tool

The dependent variable was NIAS. Unstandardized beta coefficients (*b*) are reported. R²=0.16; Adjusted R²=0.13, F_(4,99)_ = 5.03

**Table 6:** Regression analysis results showing the moderating effect (*n* = 104)

Variables	b	Std. Error	t	*p*	LLCI	ULCI
Constant	18.183	14.348	1.267	0.208	-10.283	46.648
ESRS (X)	0.554	0.535	1.036	0.208	-10.283	46.648
ORTO-11 (W)	− 0.048	0.530	− 0.090	0.020	-1.100	1.005
X x W	− 0.018	0.020	0.906	0.367	− 0.057	0.021

ESRS: The Eyuboglu Sensory Reactivity Scale

LLCI: Lower Level Confidence Interval

ULCI: Upper Level Confidence Interval

The dependent variable was NIAS. Unstandardized beta coefficients (*b*) are reported. *R* = 0.221, R²=0.049, F_(3,100)_ = 1,717

## Discussion

The literature review indicates that there are many factors influencing ARFID. In our study, we examined the sociodemographic data of individuals diagnosed with ASD and their parents, the relationship between the ARFID in ASD-diagnosed children and orthorexia in their mothers, and sensory sensitivity in children with ASD. There is no study in the literature examining the relationships between ARFID, sensory sensitivity and orthorexia in mothers of children with ASD.

In our study, all mothers were primary caregivers. Mothers play a very important role in shaping their children’s dietary habits, especially in the context of Turkish culture, where traditional family structures position the mother as the primary caregiver and decision-maker regarding nutrition. Studies show that maternal influence is important in determining children’s dietary patterns, as mothers are generally responsible for meal preparation and food selection (Hebestreit et al., 2017; Straczek et al., 2022). This responsibility goes beyond simply providing food and includes modeling eating behaviors and establishing food-related norms within the household. For example, studies have shown that mothers’ dietary behaviors, such as their own eating habits and preferences, are directly related to those of their children, suggesting that children often imitate their mothers’ dietary preferences (Groele et al., 2018; Straczek et al., 2022; Tabacchi et al., 2021).

ARFID and Pediatric Feeding Disorder (PFD) are two distinct yet overlapping diagnoses that pertain to feeding issues in children, each characterized by unique features and implications for treatment. ARFID, as defined in the DSM-5, is characterized by an avoidance or restriction of food intake that leads to significant nutritional deficiencies, weight loss, or psychosocial impairment, without the presence of body image disturbances commonly associated with anorexia nervosa (Dumont et al., 2023; Keery et al., 2019; Sanchez-Cerezo et al., 2023). The disorder manifests through three primary presentations: lack of interest in eating, avoidance based on sensory characteristics of food, and fear of aversive consequences related to eating (Dumont et al., 2023; Zickgraf et al., 2019). In contrast, Pediatric Feeding Disorder encompasses a broader range of feeding difficulties that may include ARFID but also addresses issues related to developmental delays, medical conditions, and behavioral problems that affect a child’s ability to eat (Noel, 2023; Sharp & Stubbs, 2019).

Research indicates that while ARFID is often linked to specific psychological and sensory sensitivities, PFD may arise from a combination of medical, nutritional, and behavioral factors that require a multidisciplinary approach for effective management (Greer et al., 2008; Milliren et al., 2024; Noel, 2023). For instance, children with PFD may exhibit feeding difficulties due to conditions such as gastroesophageal reflux or developmental disorders, which can complicate their eating behaviors beyond the scope of ARFID (Milliren et al., 2024; Noel, 2023). Furthermore, the treatment strategies for ARFID often focus on addressing the psychological aspects of food avoidance, while PFD interventions may prioritize improving feeding skills and nutritional intake through behavioral therapies and parental involvement (Ayoob & Barresi, 2007; Noel, 2023; Sharp & Stubbs, 2019; Taylor et al., 2019). While both ARFID and PFD involve difficulties with food intake, they differ significantly in terms of diagnostic criteria, underlying causes, and treatment approaches. In our study, we tried to make a meticulous differential diagnosis of ARFID and PFD during the case selection phase.

The literature indicates a significant overlap between ARFID and the characteristics of ASD, with neurodevelopmental disorders being more common in patients with ARFID compared to typically developing populations. It has been suggested that ASD accompanies 3–23% of ARFID cases (Kambanis et al., 2020). In a recent large cohort study of autism conducted in the United States, it was reported that 21% of ASD-diagnosed cases were at high risk for ARFID (Koomar et al., 2021). Additionally, feeding difficulties in children with ASD are five times more prevalent than in their typically developing peers (Smile et al., 2021). A hospital-based nutrition program demonstrated that 24% of 422 children with feeding difficulties also had ASD (Williams et al., 2015). The sensory sensitivities associated with ASD may contribute to the acceptance or rejection of food based on its texture, presentation, temperature, color, or smell, thus perpetuating ARFID and leading to self-imposed limitations on certain types of foods (Coglan & Otasowie, 2019). Consequently, sensory sensitivity and selective eating in children with ASD make them prone to nutritional deficiencies (Sharp et al., 2018).

Considering this, the early identification of feeding difficulties in children with ASD is essential to minimize negative health outcomes, as well as challenging mealtime behaviors and parental anxiety/stress, which can strain parent-child relationships (Smile et al., 2021). It has been shown that sensory reactivity rates can reach up to 95% in individuals diagnosed with autism (Tomchek & Dunn, 2007). In our study, we found a significant correlation between the CARS scores of children with ASD and the hypoactivity and sensory seeking subdimensions of the Sensory Reactivity Scale (*p* < 0.01). Our findings are consistent with other studies in literature that show an increase in sensory processing in relation to autism severity. A systematic review examining the relationship between sensory processing and eating behaviors in autism supported a significant relationship between taste/smell sensitivity and sensory processing with eating behaviors (Nimbley et al., 2022).

In our linear regression analysis, we found a significant regression relationship between the CARS-9 taste, smell, and touch response subdimension and the NIAS (*p* < 0.05). Our findings align with other studies that examine the relationship between taste/smell sensitivity, sensory processing, and eating behaviors (Zulkifli et al., 2022).

There is a well-documented relationship between parental and child behaviors regarding mealtime difficulties and sensory processing preferences. For instance, differences in sensory processing can contribute to caregiver stress (Schaaf et al., 2011). Sensory sensitivities and limited food preferences frequently seen in children with ASD make the responsibilities of families in nutrition management more complex (Brzoska et al., 2021; Elshafie Elnajjar, 2021). Additionally, caregiver behaviors during mealtimes can influence children’s eating behaviors (Chilman et al., 2021). Demir and Ozcan conducted a case-control study examining the feeding behaviors of children with ASD and their parents. They found that parents of children with ASD employed emotional feeding, instrumental feeding, and tolerance-controlled feeding styles more frequently (Demir & Ozcan, 2022). Emotional feeding involves providing food to a child when they are sad, unhappy, or restless, while instrumental feeding refers to rewarding a child with food when they consume an undesired food or exhibit a desired behavior. Both emotional and instrumental feeding styles are known to impact children’s food choices.

Behavioral interpretations of food refusal and selectivity highlight the role of learned behaviors and the potential consequences of these actions. For instance, children may learn to avoid foods they dislike while gaining access to preferred foods, reinforcing selective eating patterns (Cermak et al., 2010; Mari-Bauset et al., 2014). This dynamic can be influenced by caregiver responses, which may inadvertently reinforce food refusal behaviors. Research by Borrero et al. (2010) and Piazza et al. (2003) emphasizes the importance of understanding how caregiver reactions—such as providing preferred foods in response to refusal—can shape a child’s eating behaviors over time (Borrero et al., 2010; Piazza et al., 2003). Such reinforcement can lead to a cycle where the child continues to refuse less preferred foods, further narrowing their diet and potentially leading to nutritional deficiencies (Byrska et al., 2023; Marshall et al., 2014).

Moreover, it is crucial to consider medical reasons and oral motor deficits that can contribute to food refusal and selectivity. Conditions such as gastroesophageal reflux, oral aversion, or difficulty with chewing and swallowing can significantly impact a child’s willingness to eat certain foods (Amin et al., 2022; Graf-Myles et al., 2013). These medical factors may exist in isolation or in conjunction with behavioral and sensory issues, complicating the feeding landscape. For example, a child with oral motor deficits may refuse foods that require more complex chewing, leading to a reliance on softer, easier-to-eat options, which may not provide adequate nutrition (Strand, 2021; Xia et al., 2010).

In our sample, we found a significant prevalence of orthorexia symptoms among mothers of children with ASD reported through the ORTO-11 scale, which was 58%. While Varga et al. initially estimated that orthorexia occurred in 6.9% of the general population, there is no reliable measurement for its prevalence, and it may be more common among health professionals and performing artists (Varga et al., 2014).

Parents with orthorexia may impose strict dietary restrictions that prioritize perceived healthfulness over a child’s individual food preferences, potentially exacerbating food selectivity and refusal behaviors in children with ARFID (Cermak et al., 2010; Mari-Bauset et al., 2014). Children with ARFID often gravitate toward preferred foods that are typically less health-conscious and more processed, such as chicken nuggets, pizza, and fries. These foods are often favored for their consistent taste and texture, which can be particularly appealing to children with sensory sensitivities (Byrska et al., 2023; Marshall et al., 2014). However, in households where a parent has orthorexia nervosa, the availability of such foods may be limited. If these processed foods are deemed unhealthy by the parent, they may be excluded from the child’s diet, further restricting the child’s food options and potentially intensifying their food aversions (Amin et al., 2022; Graf-Myles et al., 2013).

The interplay between a parent’s orthorexia and a child’s ARFID could lead to a cycle of increased anxiety around food, as children may feel pressured to conform to their parent’s dietary ideals while simultaneously struggling with their own sensory preferences and aversions. This dynamic could hinder the child’s ability to explore new foods and develop a more varied diet, as the parent’s dietary restrictions may overshadow the child’s individual needs and preferences (Strand, 2021; Xia et al., 2010).

In our study, a significant difference was found between groups with and without orthorexia regarding the total and fear subscale of the NIAS. The fear subscale of the NIAS includes characteristics related to the fear of swallowing solid or lumpy foods, vomiting phobia, and concerns about other potential negative consequences of eating or vomiting (Bryant-Waugh et al., 2010). Our findings are consistent with other studies in the literature regarding different eating habits and food neophobia in children with ASD (Mayes & Zickgraf, 2019). Mothers with ON may excessively focus on food contents and adopt restrictive attitudes towards food types, limiting the variety of foods they offer their children based on specific diets. This situation may lead to a higher avoidant/restricted eating pattern in children with ASD.

On the other hand, no significant differences were found between groups with and without orthorexia regarding the CARS score, CARS taste, smell, and touch response suitability items, NIAS selective eating and appetite subscale, and ESRS score. Furthermore, our linear regression analysis indicated a significant regression relationship between the ORTO-11 and NIAS (*p* < 0.05).

In addition to our study findings, other factors that may influence children’s eating habits should not be overlooked. It has been suggested that mothers’ excessive concern about healthy eating may increase children’s selective eating behaviours (Ozen et al., 2024; van der Lubbe et al., 2025). It should be taken into consideration that the roles of fathers and other caregivers in the feeding process should also be examined and there is limited data in the literature on this subject (Brzoska et al., 2021; Jarman et al., 2015; Öz & Bayhan, 2021). Children with siblings may be exposed to more food diversity due to their experiences of eating together, while parents may intervene more in the diet of single children. In conclusion, since only mothers’ dietary attitudes were addressed in this study, the effects of other family members, especially fathers and siblings, on children’s eating habits were ignored. In future studies, it would be useful to address the effects of family dynamics, socioeconomic variables and children’s cognitive-emotional development on eating habits from a broader perspective.

Nutritional management of individuals with ASD requires a multidisciplinary approach that requires the collaboration of psychiatrists, nutritionists, child development specialists and psychologists (Elshafie Elnajjar, 2021). The ASD nutrition guideline published by the Ministry of Health in Türkiye draws attention to the lack of multidisciplinary approaches in this field and recommends that psychiatrists, nutritionists, child development specialists and psychologists work in coordination to increase the effectiveness of nutrition management (Sağlık Bakanlığı, 2019). The importance of interdisciplinary cooperation has been emphasized many times in international literature; it has been stated that the coordinated work of nutritionists, child psychiatrists, special education specialists, speech therapists and psychologists play a critical role in managing nutritional problems in children with ASD (van der Lubbe et al., 2024). In Türkiye, it is stated that clinical programs offering multidisciplinary nutritional support for individuals with ASD are not yet systematic enough and there is a need for development in this field (Sağlık Bakanlığı, 2019).

This study has several limitations. First, the psychiatric diagnoses of the mothers were based on self-reports, which may have led to underreporting of the frequency and variety of psychopathology. Second, because the study design focused solely on mothers, the ortorexia tendencies of fathers or other long-term caregivers were not examined. Culturally, we chose mothers because they are responsible for their children’s nutrition. However, these results do not suggest that the influence can come solely from one caregiver. Another limitation is that since this study is cross-sectional, it could not assess the eating tendencies of children at different developmental stages in relation to the orthorexia symptoms of their mothers. The current study design did not capture detailed timelines or psychological histories of mothers with ON. We indicate that future research should include in-depth interviews or longitudinal data to explore how maternal orthorexia develops over time and impacts family dynamics, particularly surrounding the ASD diagnosis. Additionally, the study included children taking psychotropic medications. Medications can have an effect on sensory sensitivity, appetite, and food selectivity.

In addressing the weaknesses identified in the manuscript concerning the focus on mothers as the primary food influencers in the context of children with ASD and ARFID, it is crucial to acknowledge the broader implications of parental roles and the multifactorial nature of restrictive eating behaviors. The emphasis on mothers, while perhaps reflective of cultural norms in Türkiye, risks perpetuating outdated stereotypes, such as the “refrigerator mother” theory, which has been discredited in contemporary autism research. This singular focus may inadvertently simplify the complex interplay of factors contributing to restrictive eating in children with ASD, leading to potential misinterpretations of the findings. In future studies, evaluating the effects of fathers as well as grandparents and other family members on children’s nutrition processes will provide a more holistic understanding of nutrition management. Parental feeding practices are dynamic and may change as children grow older, shifting from direct control over food intake to more autonomous eating behaviors. Longitudinal studies have demonstrated that mothers adjust their feeding strategies based on their children’s development and dietary responses (Jarman et al., 2015).

To enhance the manuscript’s rigor, as authors, we aimed to position it within a broader framework that highlights the multifaceted influences on restrictive eating behaviors in autism. This includes acknowledging the significant roles of both parents in shaping dietary habits, as well as the impact of individual sensory processing profiles unique to each child with ASD. Research indicates that atypical sensory processing can significantly influence food preferences and aversions, often leading to selective eating patterns that are not solely attributable to parental influence (Cermak et al., 2010; Mari-Bauset et al., 2014). Furthermore, parenting styles—ranging from indulgent to authoritative—can also play a critical role in determining a child’s dietary choices and overall nutrition (Byrska et al., 2023; Marshall et al., 2014).

To our knowledge, our study is the first to investigate the relationship between ARFID and mothers’ orthorexia in children with ASD, and we anticipate that it will contribute to the existing literature. In conclusion, our findings suggest that ARFID symptoms are predicted by maternal ON symptoms and CARS-9 scores in children with autism. However, ORTO-11 does not play a moderating role in the effect of ESRS on NIAS. In other words, the severity of ON symptoms in mothers does not have a moderating effect on the impact of sensory sensitivities in children on ARFID symptoms. ARFID is a psychiatric diagnosis that can be influenced by multiple factors. Therefore, further studies are needed to explore this relationship in more depth.
